# Rectal foreign body of a shattered glass bottle; Case report of unexpected late post-operative hemorrhage managed transanally

**DOI:** 10.1016/j.ijscr.2020.05.060

**Published:** 2020-05-30

**Authors:** Elliot Klein, Moshe Bressler, Shmuel Nadler, Michelle Shayowitz, Seth Lapin

**Affiliations:** a NYIT College of Osteopathic Medicine, Old Westbury, NY 11568, United States; b Sidney Kimmel Thomas Jefferson Medical College, Philledephia, PA 19144, United States; c Department of Gastroenterology, NYC Health + Hospitals/Coney Island, Brooklyn, NY 11235, United States

**Keywords:** Foreign body, Rectal perforation, Exploratory laparotomy, Colonoscopy, Clipping, Case report

## Abstract

**Introduction:**

Retained rectal foreign bodies are commonly implicated in patients engaging in erotic behavior. The foreign bodies vary widely, however, penetrating rectal wounds are uncommon and often complicate the retrieval of the object. The rich vascular bed of the rectal mucosa provides additional bleeding complications.

**Presentation of case:**

A 55-year-old male presented with active bleeding after intentional insertion of a glass bottle into the rectum which shattered. Partial retrieval via proctoscopy was followed by an exploratory laparotomy with a diverting colostomy, mucous fistula, and presacral drainage. Postoperative course was complicated by severe hematochezia. Colonoscopy was performed in the operating room found actively bleeding ulcers at sites of previously lacerated mucosa; one pulsating, protruding, vessel was visible. Clips were placed over the vessel with cessation of bleeding. Barium enema at three months follow revealed no leaks allowing for reversal of the colostomy.

**Discussion:**

This case of operative retrieval of a rectal foreign body is unique because it displays deviation from commonly used algorithms are periodically needed to optimize patient recover.

**Conclusion:**

The utilization of minimally-invasive colonoscopy prior to additional surgical intervention in post-operative rectal bleeding following a rectal foreign body retrieval may improvie patient recovery time, functionality, and long-term outcomes.

## Highlights

• Colonoscopy post perforating rectal injury may be employed to evaluate and treat bleeding.
• DRE should be cautioned when suspecting hazardous material in the colorectal tract.
• Mobilization of foreign bodies must be determined in a case by case basis.

## 1 Introduction

Retained rectal foreign bodies are an increasingly common presentation requiring emergent surgery [1]. The most common cause of foreign body insertion is due to eroticism followed by assault, prostate massages, relief of constipation, and concealment of illegal objects [1]. Men comprise more than two thirds of the patient population implicated in retained foreign bodies, with the most common ages being between twenty and thirty years old [2]. However, patients as old as 90 have been reported as well [2]. The object can often be retrieved at bedside. However, in the setting of a retained foreign body, especially one complicated by bleeding, perforation, or peritonitis, open surgery or laparoscopic assisted transanal retrieval is often needed [3]. Careful investigation and a high index of suspicion is required as patients are often reluctant to share the etiology of their presentations. Here, we present a 55-year old male who inserted a glass bottle which subsequently shattered resulting in mucosal lacerations and a rectal perforation leading to a complicated retrieval process and post-operative course. The case has been reported in line with SCARE criteria and guidelines [4].

## 2 Presentation of case

A 55-year-old male presented to the Emergency Department (ED) with complaints of rectal bleeding for a three-hour duration. Initially, the patient stated that he had fallen down, which resulted in rectal bleeding, however, after further inquiry he admitted to placing a glass bottle in his rectum. He stated he had intentionally placed a glass bottle as he engaged in autoerotic behavior. After removing the glass object, he noticed the top half of the bottle was missing and retained inside his rectum. He developed mild rectal pain and bright red blood per rectum, but denied dizziness, weakness, or palpitations.

**Fig. 1:**
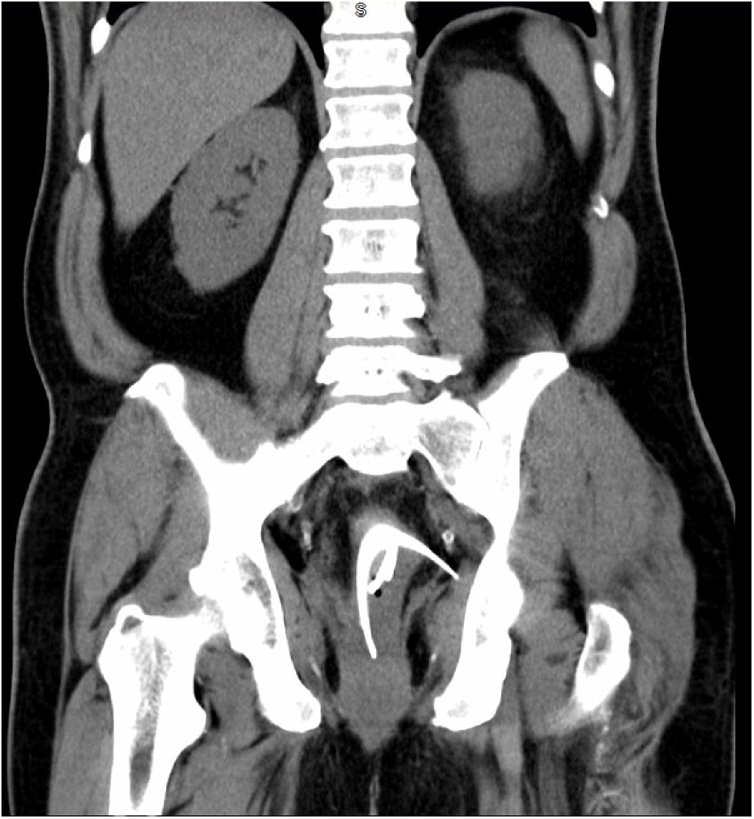
Coronal CT revealing multiple fragments of glass. One portion of the glass appeared to be traversing the inferior left lateral rectal wall, which was suspicious for perforation, however, no free air was present.

On examination, no peritoneal signs were noted. Digital rectal examination (DRE) revealed no signs of perianal lesions, sphincter lacerations, or palpable foreign bodies. Initial labs displayed a reduced, but stable, hemoglobin and hematocrit at 11.7 and 34.1, respectively. Computed Tomography (CT) of the abdomen and pelvis revealed multiple radiopaque foreign bodies in the distal rectosigmoid measuring up to 14 cm in length, as well as presacral edema (Fig. 1). In correlation with the patient's history, it was assumed to be a glass bottle broken into multiple pieces. One portion of the glass appeared to be traversing the inferior left lateral rectal wall, which was suspicious for perforation, however, no free air was present. The patient was consented for exam under anesthesia, sigmoidoscopy, presacral drainage, exploratory laparotomy, and colonic diversion to retrieve the foreign body and manage possible pelvic sepsis.

**Fig. 2:**
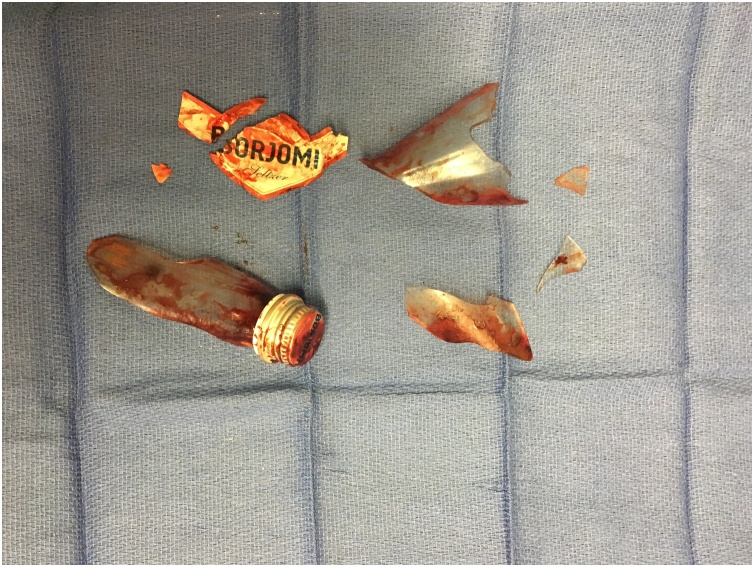
Multiple shards of glass bottle removed from the distal sigmoid and rectum.

Patient was placed in lithotomy position under general anesthesia. A Lone Star Retractor was employed with large size proctoscopy, displaying several large sharp broken pieces of glass with abundant blood and clot formation. Glass which was readily dislodgeable via a transanal approach was retrieved while larger perforating pieces were carefully mobilized proximally to ease retraction via laparotomy. An exploratory laparotomy was performed, the sigmoid was divided, and additional foreign bodies were removed from the distal sigmoid. Flexible sigmoidoscopy confirmed complete foreign body removal with three subcentimeter deep lacerations with resultant perforations in the rectal wall, mostly contained in the proximal rectum. A diverting colostomy and mucous fistula through the abdomen were created, and presacral drainage was performed. In total, five pieces of glass were removed along with an intact bottle cap and label (Fig. 2).

Post-operatively, the patient was monitored in the Surgical Intensive Care Unit (SICU) and was recovering well. On post-op day seven, large volume rectal and mucus fistula bleeding was noted in conjunction with severe hypotension, tachycardia, change in mental status and a drop in hemoglobin to 5.8. Rapid transfusion protocol was activated with administration of two units of packed red blood cells (PRBCs) and an appropriate response to a hemoglobin of 8.5 was observed. CT-angiogram displayed a hyperemic rectum with pericolonic inflammatory changes consistent with proctitis, without evidence of active contrast extravasation. Repeat episodes of high volume bloody rectal evacuations continued to occur over the following 12 hours, resulting in resuscitation with three additional units of PRBCs and two units of fresh frozen plasma (FFP).

**Fig. 3:**
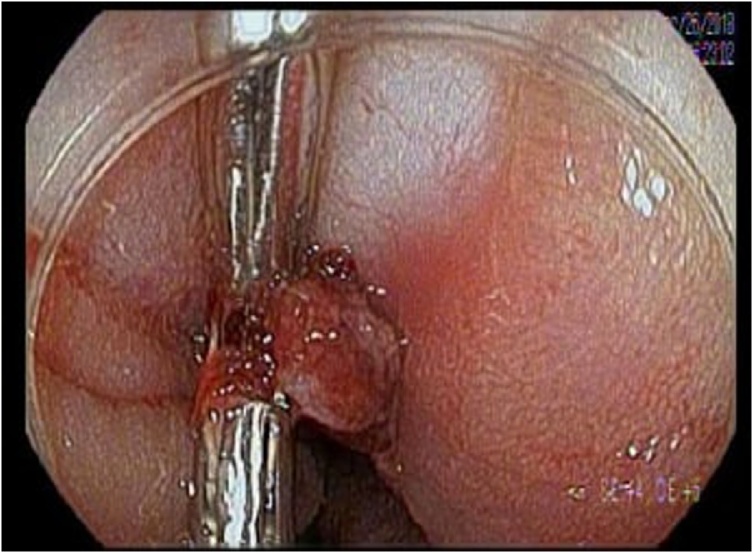
Post-operatively, at a laceration site, an ulcer approximately 7-cm from the anal verge contained an actively bleeding vessel successfully treated with two hemostatic clips.

Colonoscopy was performed in the operating room. Copious blood was noted in the distal rectum with three ulcers located at sites of previously lacerated mucosa. Blood was noted in the distal rectum with one ulcer, approximately seven cm from the anal verge, contained a pulsating, protruding, visible vessel actively bleeding. Two clips were successfully placed over the vessel with no further episodes of bleeding (Fig. 3). At the one-month follow-up, the patient was found stable and asymptomatic, with no rectal pain or bleeding. At three months the patient was doing well. A barium enema was performed with no leaks noted followed by subsequent reversal of the colostomy.

## 3 Discussion

Retained rectal foreign bodies, most often the result of anal eroticism, have become a more frequent yet continuously underreported occurrence. Recent literature has indicated an increasing incidence of retained rectal foreign bodies resulting in impaction and resultant complications [5]. Patients are often reluctant to report the presence of a foreign body, which ultimately leads to a delay in treatment due to complaints of obscure pain comprised of strange etiologies [6]. Various algorithms use a step-up approach and are commonly utilized for the management of rectal foreign bodies. However, a retained foreign body is not commonly the cause of a surgical emergency [7]. When suspecting a patient with a retained rectal foreign body, a thorough history and physical exam are paramount to ensuring proper management and outcomes for the patient. A CT scan or abdominal x-ray is routinely necessary as well. If the history indicates an impacted blunt object, bedside retrieval can often be attempted via transanal approach [3,8]. Endoscopic techniques, perianal nerve blocks, Valsalva maneuvers, obstetric vacuums, clamps, and foley catheters have been employed to facilitate bedside removal. However, in the presence of free air, peritoneal signs, sharp objects or the inability to retrieve the object at bedside, surgical intervention consisting of either laparoscopic removal or laparotomy with a colostomy should be considered without delay [8]. Approximately 55% of cases in which foreign bodies have reached the proximal rectum have escalated to a laparotomy, while only 24% of objects localized to distal rectum have required open surgical intervention [9].

Two of the most important components of the management of traumatic rectal injury include colostomy and presacral drainage [10]. Colostomy diversion allows for uninterrupted healing of the laceration and perforation without the presence of stool passing over the injury site. Presacral drainage has been shown to reduce hematoma and abscess formation [10]. Following removal of a foreign body, specifically in those requiring surgical intervention or resulting in suspected rectal perforation, a proctosigmoidoscopy or colonoscopy is conducted to assess mucosal damage as well as confirm complete removal. Complications occurring after the initial surgery, such as rectal bleeding, are rare and are normally handled by a return to the OR in an attempt to control the source.

Our case presents several interesting findings that warrant exploration. The patient reported that upon placement of the bottle in his rectum the bottle was full of soda water. Continuous shaking and motion of the bottle caused the bottle to explode intrarectally. The force of the bottle exploding drove shards of glass through distal rectosigmoid. During the retrieval of the bottle, it was noted that the cap of the bottle was still intact, supporting the patients recount. The series of lacerations and perforations that followed resulted in ulcerations of localized mucosal vessels leading to secondary hemorrhage and a complicated postoperative course.

The use of colonoscopy after a perforating rectal injury is not well discussed in literature. Diffuse rectal bleeding resulting in a precarious drop in hemoglobin is a life-threatening postoperative emergency. In this case, the bleeding was unable to be localized to a specific vessel on angiography but was clearly occurring within the sigmoid or rectal vault and not intra or retroperitoneal. Utilization of colonoscopy to evaluate and stop the source of bleeding allowed for avoidance of an additional operation and a decrease in recovery time. Localization of bleeding via endoscopy allowed for clips to be placed over the bleeding vessel, resulting in cessation of the hematochezia.

Additionally, the commonly utilized step-up algorithmic approach often includes a digital rectal exam as part of the initial physical examination in order to localize and palpate the foreign body [1,3,8]. However, it is important to note that the use of a digital rectal examination may be in fact hazardous to the examiner in cases like ours. Multiple shards of glass in several planes presents a danger to palpation of the rectal vault even if dilation of the anal sphincter is achieved or an anoscope is employed. Few cases can be found that present with sharp hazardous material at an undetermined height in the colorectal tract. It is paramount to refrain from conducting a DRE until the nature of the foreign body is either verbally confirmed by the patient or via imaging. Often this can be further complicated by a patient reluctant to share a detailed history due to the nature of the injury.

Lastly, retrieval of the foreign body via surgical intervention often requires distal milking of the object towards the anus [3,8]. In our case, we felt that milking the retained shards downwards would further damage the mucosa. Instead, glass that was readily transanally mobilized in line with its initial trajectory was removed in that fashion. Larger shards, or shards that were pointed inferiorly and thus better accessed via the proximal sigmoid, were milked proximally towards the divided sigmoid and retrieved through the laparotomy incision. Intraoperative judgement in cases involving sharp foreign bodies that are impacted at disadvantageous trajectories must be employed in order to determine the safest route of extraction. Additionally, the skill of the surgeon must be taken into account. Distal vs proximal mobilization of the foreign body must be determined in a case by case basis depending on trajectory, impaction, and safest way of removal to minimize further damage.

## 4 Conclusion

Retained rectal foreign bodies can present as challenging clinical scenarios especially if traumatic damage to the colorectal tract is present. Common practice utilizes a step-up approach in determining the next best step in management with few cases requiring emergent surgery. When indicated, surgical intervention ranges between laparoscopic assisted transanal retraction and exploratory laparotomy. On initial history and physical exam care must be given to attempt to determine the nature of the object prior to digital rectal examination. Hazardous material present in the rectum may injure the examiner. In cases requiring laparotomy mobilization of the object proximally towards the divided sigmoid or transanally must be decided intraoperatively in order to minimize further damage to the tract. Although rare, complications that arise after surgery should be considered for colonoscopic evaluation prior to additional surgical intervention. Ultimately, improving patient recovery time, functionality, and long-term outcomes should remain a priority while assessing a patient with traumatic injury due to a retained rectal foreign body.

## Conflict of interest

The authors declare no conflict of interest to disclose.

## Funding

The authors declare no competing financial interest to disclose and received no funding.

## Ethical approval

This case received Academic Review Board approval as well as IRB exemption via BRANY IRB Institutional Services as it was not experimental in nature, provided minimal to no risk to the patient, and contains no-identifiable data about humans with consent received from the patient.

## Consent

Informed consent was obtained from the patient to use his information and studies anonymously.

## Author contribution

EK: Wrote the case, gathered information and research.

MB: Case write up and image harvesting.

SN: Case editing formatting.

MS: Edited and gathered sources.

SL: Gastroenterology attending present for care of patient. SL had full access to all of the information in the case and takes full responsibility for the integrity of the data and the accuracy of the information. All authors read and approved the final manuscript.

## Registration of research studies

NA.

## Guarantor

Seth Lapin had full access to all of the information in the case and takes full responsibility for the integrity of the data and the accuracy of the information. All authors read and approved the final manuscript.

## Provenance and peer review

Not commissioned, externally peer-reviewed.
